# Eco-Friendly Polysaccharides as Moisture Retainers: Influence on Humic Acid Colloidal Stability in Model and Natural Systems

**DOI:** 10.3390/molecules30173618

**Published:** 2025-09-04

**Authors:** Gleb N. Trishkin, Maria G. Chernysheva, Natalia A. Kulikova, Gennadii A. Badun

**Affiliations:** 1 Department of Chemistry, M.V. Lomonosov Moscow State University, Leninskiye Gory 1, Moscow 119991, Russia; trishkin.gleb1@gmail.com (G.N.T.); badunga@my.msu.ru (G.A.B.); 2 Department of Soil Science, M.V. Lomonosov Moscow State University, Leninskiye Gory 1-12, Moscow 119991, Russia; kulikova-msu@yandex.ru

**Keywords:** hyaluronic acid, humic acids, carboxymethyl cellulose, co-adsorption, liquid–liquid interface, wetting

## Abstract

Hyaluronic acid and carboxymethyl cellulose are eco-friendly polysaccharides known for their excellent moisture retention properties, making them suitable components of agricultural fertilizers. On the other hand, humic acids exhibit surface-active properties, suggesting their potential to replace synthetic surfactants in agricultural applications. Naturally, the interaction between polysaccharides and humic substances influences their colloidal and chemical behavior. The mutual interactions between humic acids and these polysaccharides were examined at immiscible liquid interfaces and on plant leaf surfaces using radiotracer analysis and tensiometry (pendant drop and sessile drop methods). The results indicate that humic acids achieve optimal adsorption at a hyaluronic acid concentration of 30 g/L, regardless of molecular weight. In contrast, carboxymethyl cellulose reduces the surface activity of humic acids. Additionally, a combined solution of humic acids and hyaluronic acid improves the wetting efficiency of wheat leaves compared to individual solutions. However, humic acids showed minimal impact on the absorption or systemic distribution of hyaluronic acid within the plant.

## 1. Introduction

Recently, compounds of biological origin have aroused great interest in the food industry as promising adjuvants for various foliar nutrition agrochemicals [1]. Foliar nutrition is considered to be one of the most common strategies for delivering the necessary microelements to plants, resulting in an increase in both the crop yield and its quality [2]. The main issue in foliar nutrient application is the hydrophobic waxy cuticle, covering plant foliage surfaces and presenting an effective barrier to the penetration of exogenous chemicals. To overcome this problem, adjuvants are usually added to nutrient formulations, and the most common way to improve the interaction of spray droplets and the plant leaf surface is through the use of surfactants lowering the surface tension of nutrient spray solutions and, as a consequence, lowering the contact angle of droplets on the plant leaf surface. The latter is considered to increase the rate of uptake of foliar-applied fertilizers [3].

Among the natural substances with surface activity that are important for agriculture, humic acids (HA) are the most well-known. They represent the dominant form of natural organic matter in the environment, with a ubiquitous presence in marine, aquatic, soil, and sedimentary ecosystems. Their ability to enhance uptake has been successfully exemplified by iron nanoparticles [4]. An additional advantage of HA is their own biological activity, allowing for the application of commercial humic products in agriculture [5].

Along with surfactants, moisture retainers (humectants) are also effective adjuvants due to their ability to prolong the time taken for nutrient absorption by leaves [3]. Examples of naturally occurring substances with pronounced water-retaining properties include polysaccharides such as hyaluronic acid and carboxymethyl cellulose (CMC). Polysaccharides are abundant resources that are biodegradable and eco- and environmentally friendly. Polysaccharides show a high potential for application in nanobiotechnology and agriculture, particularly as hydrogels that enable the high sorption of ingredients and gradual release [6,7,8,9], or as solutions for preservation and moisture retention [10,11,12,13,14].

Hyaluronic acid is a naturally occurring non-sulfated glycosaminoglycan with a high moisture retention capacity as well as elicitor activity for disease prevention in plants [15] and a stabilizing effect on nanoparticles [16,17]. While glycosaminoglycans are well-known for their remarkable water-retention capacity, their interfacial behavior, especially in complex biological environments containing other biopolymers, remains poorly understood and poses significant research challenges [18].

CMC is a linear polysaccharide with valuable applications in agriculture due to its water-retention, film-forming, and biodegradable properties. Being mixed with other polymers, CMC forms eco-friendly films that suppress weeds and retain soil moisture [19,20].

Both polymers contain carboxyl groups capable of interacting with HA functional groups (–OH, –NH_2_, etc.) via ionic bonds, hydrogen bonds, or complexation. Additionally, their hydrophilic nature and high moisture retention capacity allow for the modification of HA hydration shells, thereby influencing aggregation. As carbohydrate-based polymers, hyaluronic acid and CMC act as colloidal stabilizers by increasing medium viscosity, introducing electrostatic repulsion (due to their anionic charges), and reducing surface tension at interfaces, thereby altering HA adsorption dynamics.

If we use a combination of moisture-retaining carbohydrates and HA as a replacement for synthetic surfactants in crop treatment sprays, these substances will naturally influence each other’s behavior. Therefore, it is essential to optimize this mixture’s composition to achieve maximum surface activity and penetration efficiency. That was the purpose of this study.

In the present research, we compare the effects of hyaluronic acid and CMC on the colloidal behavior of HA. Hyaluronic acid and CMC were chosen as moisture-retaining compounds with potential agricultural applications.

## 2. Results and Discussions

### 2.1. The Effect of Moisture-Retaining Compounds on the Hydrophobic and Surface-Active Properties of Humic Acids

**Figure 1:**
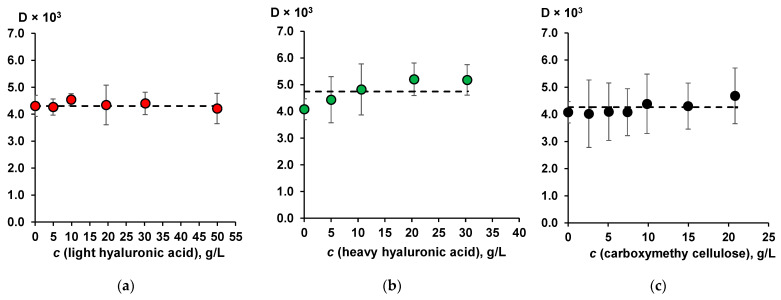
The dependence of the HA distribution coefficient in the aqueous–toluene system on the moisture-retaining compound concentration: (**a**) light hyaluronic acid; (**b**) heavy hyaluronic acid; (**c**) CMC. HA concentration is 30 mg/L, pH is 6.8. Bars represent the standard deviation (n = 3). The dashed line represents averaged values for *D* (see text) equal to 4.4, 5.2 and 4.4 for light hyaluronic acid (**a**); heavy hyaluronic acid (**b**); and CMC (**c**), respectively.

The first step was to determine the distribution coefficient (*D*) of HA between the aqueous and toluene phases using the scintillation phase method [21,22,23]. Figure 1 shows *D* of HA as a function of moisture-retaining compound concentration. *D* of the compound was calculated as a ratio between its equilibrium concentrations in the toluene and aqueous phases (*c_aq_*).

(1) $$D=\frac{c_{org}}{c_{aq}}$$

The HA distribution coefficient does not depend on the concentration of light hyaluronic acid and CMC and its average value is equal to (4.4 ± 0.4) × 10^−3^. The addition of heavy hyaluronic acid had no statistically significant effect on the distribution coefficient of HA. The average value of *D* was (5.2 ± 0.6) × 10^−3^. Note that the *D* values were not affected by the moisture-retaining compound concentration. Therefore, the data obtained were combined into a single corresponding statistical sample for each moisture-retaining compound. When analyzing the effect of light and heavy hyaluronic acid and CMC, the obtained samples were compared with each other and no significant difference was found between them.

**Figure 2:**
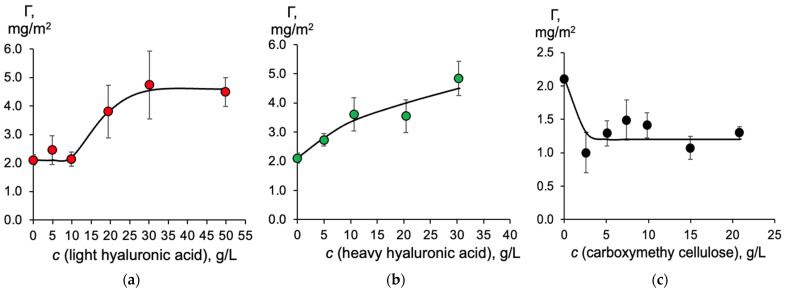
The dependence of the HA surface concentration (Γ) at the aqueous–toluene interface on the moisture-retaining compound concentration: (**a**) light hyaluronic acid; (**b**) heavy hyaluronic acid; (**c**) CMC. HA concentration is 30 mg/L, pH is 6.8. Bars represent the standard deviation (n = 3).

An important characteristic that can be directly measured using the scintillation phase method is the surface concentration of a tritium-labeled compound at the liquid–liquid interface. Figure 2 shows the HA surface concentration (Γ) dependence on the moisture-retaining compound concentration.

The surface concentration of free HA at the aqueous–toluene interface is 2.2 ± 0.3 mg/m^2^. This value remains constant when light hyaluronic acid is added up to a concentration of 10 g/L. However, the HA surface concentration doubles when the light hyaluronic acid concentration reaches 20 g/L, and after that it remains practically unchanged with further increases in light hyaluronic acid concentration. Note that light hyaluronic acid does not form a gel in the bulk of the aqueous phase at concentrations ranging from 5 to 50 g/L, while heavy hyaluronic acid forms a gel when its concentration exceeds 10 g/L. This fact should be taken into account when considering its effect on the behavior of HA in a two-phase system. The addition of heavy hyaluronic acid to HA results in a monotonous increase in the surface concentration, which reaches a value of around 5 mg/m^2^ at a hyaluronic acid concentration of 30 g/L. This HA surface concentration is similar to the maximum value obtained in the presence of light hyaluronic acid.

The addition of CMC results in a twofold decrease in the surface concentration of HA. It reaches 1.2 ± 0.2 mg/m^2^ when the CMC concentration is 2.5 g/L and it does not change with the increase in concentration. The difference in the influence of hyaluronic acid and CMC can be explained by the following reasons. Surface active aggregates can be formed between hyaluronic acid and HA via the formation of hydrogen bonds (the presence of -OH and -COOH in both polymers) as well as through hydrophobic interactions between the nano-polar fragments of hyaluronic acid and HA.

CMC reduces the surface concentration of HA because, unlike hyaluronic acid, it contains almost no hydrophobic groups and, therefore, is incapable of forming mixed aggregates with HA. Due to its high charge density on carboxyl groups, CMC forms a strong polyanion that, upon adsorption at the interface, reduces the local concentration of HA through electrostatic repulsion. The other reason is the formation of viscous barriers by rigid linear chains of CMC that obstruct the diffusion of HA to the interface.

Thus, hyaluronic acid becomes the preferred moisture-retaining agent when combined with humic acids to create an eco-friendly hydrogel as a substitute for surfactants. Therefore, we considered the effect of HA on the colloidal properties of hyaluronic acid and tested its properties when wetting the surface of a wheat leaf.

### 2.2. The Effect of Humic Acids on the Hydrophobic and Surface-Active Properties of Hyaluronic Acid

**Figure 3:**
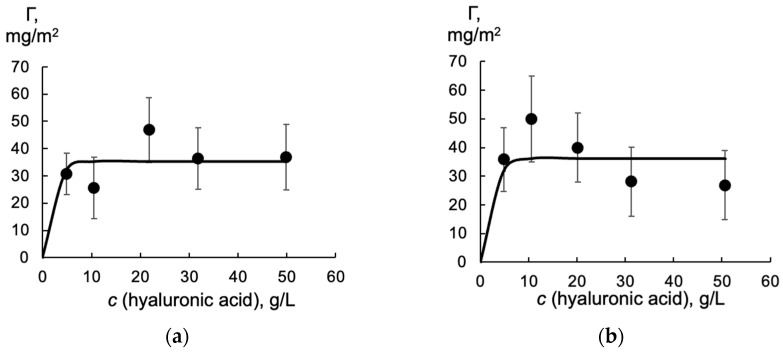
The dependence of the surface concentration of light hyaluronic acid at the aqueous–toluene interface on its concentration in the bulk of the aqueous phase. (**a**) Free hyaluronic acid; (**b**) in the mixture with HA. HA concentration is 30 mg/L, pH is 6.8. Bars represent the standard deviation (n = 3). The solid lines show the trend in adsorption changes and are drawn to guide the eye.

In this experiment, we used tritium-labeled “light” hyaluronic acid, along with non-radioactive HA. The distribution coefficient of light hyaluronic acid in the aqueous–toluene system was found to be (1.3 ± 0.5) × 10^−3^, independent of the HA concentration. The surface concentration of light hyaluronic acid at the aqueous–toluene interface was approximately 40 mg/m^2^, and this also showed no dependence on the presence of HA (Figure 3).

High values of the hyaluronic acid surface concentration indicate the formation of a poly layer at the liquid–liquid interface, which is typical of polysaccharides that stabilize oil–water emulsions. This occurs through the formation of a gel-like layer at the boundary, as seen in systems containing hyaluronic acid [24,25,26]. Note that the presence of 30 mg/L of HA does not significantly influence the surface concentration of hyaluronic acid. The data presented in Figure 3 at a level of 0.05 do not differ significantly.

### 2.3. Peculiarities of the Interfacial Changes at the Aqueous–Toluene Interface When Hyaluronic Acid and HA Are Present

**Figure 4:**
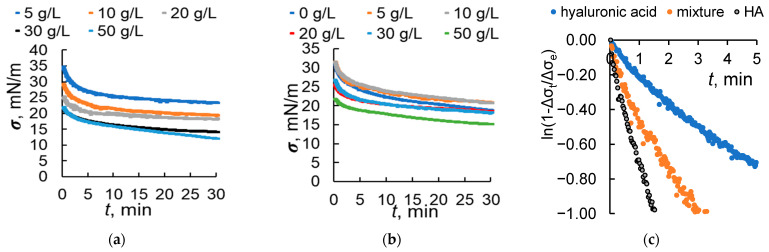
Time dependences of the interfacial tension at the aqueous–toluene interface. (**a**) Light hyaluronic acid; (**b**) HA + light hyaluronic acid (HA concentration is 30 mg/L, pH is 6); (**c**) the initial part of the dependence of surface tension on time is shown in the coordinates of Equation (2) for hyaluronic acid (30 g/L), HA (30 mg/L) and their mixtures at these concentrations.

Figure 4 shows the time dependence of the interfacial changes at the aqueous–toluene interface for systems that contain HA, light hyaluronic acid or its mixture. The HA concentration in the mixture with hyaluronic acid is 30 mg/L.

It can be seen that the interfacial tension decreases more sharply in the case of a mixture containing 30 mg/L of HA and 30 g/L of light hyaluronic acid. Notably, this ratio corresponds to the constant surface concentration of humic acids in the presence of hyaluronic acid (Figure 2a). This value is controlled by the concentration of hyaluronic acid at 50 g/L. The rate of the interfacial tension changes can be described by the following equation [27]:

(2) $$\ln\left(1-\frac{\Delta \sigma_{t}}{\Delta \sigma_{e}}\right)=-kt$$

where Δ*σ_t_* and Δ*σ_e_* are the surface tension changes at time *t* and in the equilibrium state, respectively, and *k* is the rate constant that can be estimated from the slope of ln(1 − Δ*σ_t_*/Δ*σ_e_*) versus *t*, and when *t* <100 s, the adsorption is controlled by diffusion. For free hyaluronic acid (30 g/L), HA (30 g/L) and the HA–hyaluronic acid mixture at the same concentrations, the observed diffusion rate constant was (3.3 ± 0.1) × 10^−3^ s^−1^, (1.1 ± 0.1) × 10^−2^ s^−1^ and (6.8 ± 0.1) × 10^−3^ s^−1^, respectively. Such values of the rate constant explain the surface tension control with HA in the first minutes, while the scintillation phase of the experiment indicates an excess of hyaluronic acid at the interface after one week of storage.

Based on these results and previous data [28], an aqueous solution of the following composition was selected for further plant experiments: 0.028 M of phosphate buffer, 1 M of urea, 30 mg/L of HA, and 30 g/L of light hyaluronic acid.

### 2.4. Wetting the Surface of a Wheat Leaf with Solutions of Hyaluronic Acid and Humic Acid

Wetting the leaf surface is a crucial stage to ensure the penetration of agrochemicals into the plant during foliar fertilization. One of the factors influencing this process is the composition of the leaf surface wax. In wheat leaves, the wax composition may vary depending on the organ’s age [29,30,31]. Therefore, we analyzed both leaves separately (the first leaf and the second leaf) when studying their wetting properties. A detailed analysis of humic substances applied for foliar treatment shows positive effects on plant development and crop yield across a wide range of agricultural crops [32].

The contact angle (CA) is used to measure the degree of wettability of a leaf by a corresponding solution [33,34]. Depending on wettability, surfaces can be categorized as superhydrophilic ones, where a fluid will spread and cover a large surface area, with a CA ranging from 0° to 10°. Surfaces with a CA between 10° and <90° are termed hydrophilic. Surfaces with a CA between >90° and <150° are hydrophobic, and finally, a surface is defined as superhydrophobic when it demonstrates a static CA >150° [35,36,37]. Most terrestrial plant surfaces are classified as hydrophobic or superhydrophobic [36,38,39]. Since urea improves wetting and enhances water penetration into leaves [40], 1 M of urea in phosphate buffer was used as a control for the wetting study. The same urea concentration was used in both tested solutions containing HA and light hyaluronic acid.

**Figure 5:**
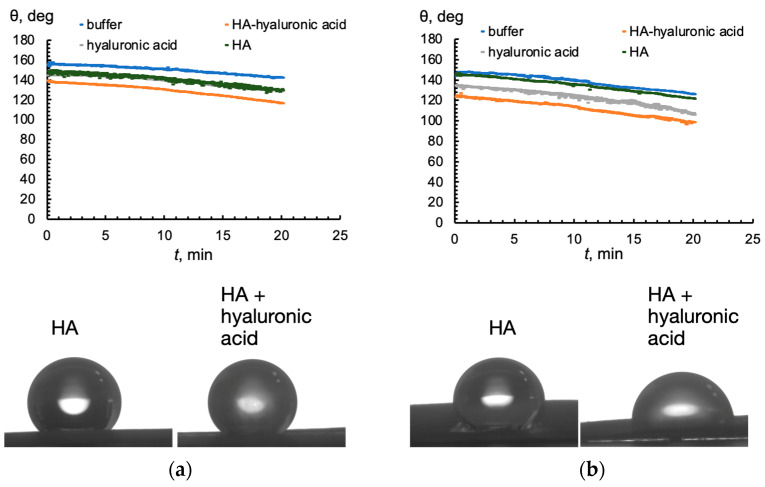
The time-dependent wetting contact angle (in degrees) of the wheat leaf surface treated with solutions of humic acids, hyaluronic acid, and a mixture of humic and hyaluronic acids. (**a**) First leaf; (**b**) second leaf. HA concentration is 30 mg/L, hyaluronic acid concentration is 30 g/L, pH is 6.8.

Figure 5 shows the time-dependent wetting contact angle of a wheat leaf treated with HA, light hyaluronic acid and with their mixture. The study used 8-day-old wheat seedlings, each with two leaves, and wettability was analyzed separately for each leaf.

In the case of the control solution, the values of CA were 160° and 150° for the first and second leaves, respectively, indicating a superhydrophobic surface of the wheat leaf [33]. The addition of free HA or free hyaluronic acid results in a slight decrease in the contact angle in both cases. This result correlates with the interfacial tension at the aqueous–toluene interface: free HA and hyaluronic acid reduce interfacial tension from 35 mN/m for free buffer to 18 ± 2 mN/m and 14 ± 2 mN/m, respectively. The wetting effect was particularly strong for the HA–hyaluronic acid mixture. Hyaluronic acid facilitates the accumulation of HA at the hydrophilic–hydrophobic interface. The high diffusion rate constant of HA contributes to its rapid initial adsorption, thereby enhancing leaf surface wetting. These results explain the synergistic effect of humic substances and hyaluronic acid in wetting the surface of wheat leaves. The measured wetting contact angles were below 140° for the first leaf and below 120° for the second leaf. Note that the values of CA obtained in our study are higher than those reported in Ref. [4], where 21-day-old wheat seedlings were treated with solutions containing iron (hydr)oxide nanoparticles and humic substances. The reason for this difference might be the concentration of humic substances, which was close to 7 g/L in the cited study, whereas in our case, it was only 30 mg/L. We used lower concentrations of HA based on our previous study, which demonstrated that the adsorption of humic substances on hydrophobic surfaces reached a constant value starting from 20 mg/L, regardless of their origin [23].

The results show that a combined solution of HA and hyaluronic acids in concentrations of 30 mg/L and 30 g/L, respectively, demonstrates superior wetting properties for both leaves compared to single-component solutions. This synergistic effect allows for a more uniform distribution of foliar fertilizer, which is particularly beneficial for hydrophobic crop species. The enhanced droplet spreading and retention achieved through this formulation can significantly reduce the amount of agrochemicals used.

### 2.5. The Role of HA in Hyaluronic Acid Uptake by Leaves

The leaf wettability mechanism involves three key processes: collection, adsorption, and transportation. Therefore, the adsorption of moisture-retaining agents and their potential transport within the leaf are critically important. Table 1 presents the mass content of hyaluronic acid in different leaf sections.

The results indicate that the presence of hyaluronic acid has little to no effect on either hyaluronic acid absorption or its distribution. Furthermore, it was demonstrated that once hyaluronic acid penetrates the leaf, it remains localized at the application site without diffusing into other tissues or organs.

Therefore, in the case of polymeric compounds that can be considered as moisture-retaining agents for crops, HA cannot be regarded as an adjuvant in agricultural sprays, replacing artificial surfactants that significantly improve penetration into the plant. As follows from Table 1, a slight increase in the uptake of hyaluronic acid indirectly confirms the weak interaction between hyaluronic acid and HA that we observed in the previous paragraphs regarding changes in the colloidal behavior of HA, particularly the increase in HA adsorption at the liquid–liquid interface and the decrease in the contact angle when wetting the wheat seedling. We can therefore propose using these natural polymers as promising multifunctional adjuvants that could serve as a sustainable alternative to synthetic surfactants in agriculture. While these biopolymer-based solutions offer clear environmental advantages, their practical implementation requires (1) optimizing crop-specific formulations, and (2) developing economically viable production methods to ensure commercial feasibility.

## 3. Materials and Methods

### 3.1. Materials

Potassium humate “Sakhalin” with a humic acid content of 80% was a product of “Green Island” (Moscow, Russia) and used as received. Two hyaluronic acid samples in the form of sodium salt were purchased from “Shandong AWA Biopharm” (Binzhou, Shandong, China) and “NaturFree” (Buchs, Switzerland). Carboxymethyl cellulose in the form of sodium salt was a product of “Sigma” (St. Louis, MO, USA); its molecular weight was about 150 kDa according to the results obtained using the viscosimetric method.

Hyaluronic acid was analyzed using size-exclusion chromatography (SEC) to determine its molecular weight and polydispersity index (PDI). SEC was performed using an Ultrahydrogel 1000 column (Waters) pre-calibrated with dextran standards, and potassium phosphate buffer (pH 7.7) was used as the mobile phase. It was found that the “NaturFree” (Switzerland) hyaluronic acid sample had the following characteristics, Mn = 1.5 MDa, Mw = 1.9 MDa and PDI = 1.3, while the “Shandong AWA Biopharm” (China) sample had Mn = 25 kDa, Mw = 54 kDa and PDI = 2.2.

### 3.2. Radiotracer Used in Studying Polymers’ Behavior at the Interfaces: Liquid–Liquid and the Leaf of a Wheat Seedling

Tritium-labeled humate and hyaluronic acid were obtained by means of the tritium thermal activation method [21,22,23]. The specific activity of the labeled compounds was 110 and 39 GBq/g for humate and hyaluronic acid, respectively.

The scintillation phase experiment was performed in a phosphate buffer (pH 6.8) of the following composition: Na_2_HPO_4_ × 12H_2_O (3 g/L), KH_2_PO_4_ (2.5 g/L). The experiment was performed according to the standard procedure previously described [23]. Briefly, a 1 mL portion of tritium-labeled compound solution of known contrition and specific radioactivity (*a*_sp_) was added to the scintillation vial, followed by the addition of 3 mL of toluene-based scintillator (8 g/L of 2.5-diphenyloxazole in toluene). The two-phase systems were stored at 25 °C to equilibrate [23], and then, using a liquid scintillation spectrometer (RackBeta 1215, LKB Wallac, Turku, Finland), we measured the count rate of tritium beta-radioactivity of the whole system (*I*), the separated part of the organic phase, and the residual two-phase system after sampling an aliquot from the organic phase. Values of the tritium-labeled compound concentration in the bulk of the organic phase (*c_org_*) and at the liquid–liquid interface (Γ) were calculated as follows:

(3) $$c_{org}=\frac{I_{1}}{60\varepsilon V_{1}a_{sp}}$$

(4) $$\Gamma =\frac{I-\frac{I_{1}}{V_{1}}V}{\frac{1}{2}\varepsilon Sa_{sp}60}=\frac{I_{2}-\frac{I_{1}}{V_{1}}V_{2}}{\frac{1}{2}\varepsilon Sa_{sp}60}$$

Here, V is the volume of the organic phase, indexes 1 and 2 refer to the aliquot of the organic phase and the system after removing a bit of the organic phase; S is the area of the interface; and ε is the registration efficiency of tritium radiation in the bulk of the xylene scintillator.

**Figure 6:**
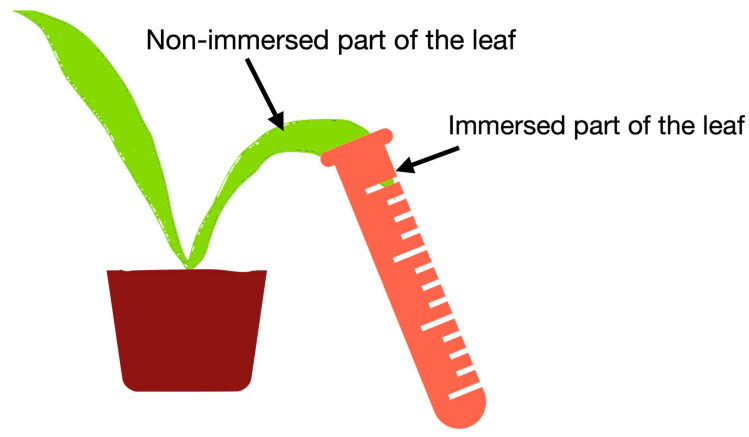
Scheme of foliar uptake of [^3^H]hyaluronic acid by wheat seedlings.

Tritium-labeled light hyaluronic acid was also used to study its foliar uptake by common wheat *Triticum aestivum* L. cv. Akhtyrchanka. In these experiments, 14-day wheat seedlings were used. The seeds were germinated in the dark at 24 °C for 24 h. Then, the imbibed seeds with the visible germinal root were transferred into 1 L polyethylene tanks containing perlite supported with Knop nutrient solution (0.14 g/L KH_2_PO_4_, 0.1 g/L KCl, 0.14 g/L KNO_3_, 1.42 g/L MgSO_4_·7H_2_O, 4.88 g/L Ca(NO_3_)_2_·12H_2_O, pH 5.5) and cultivated in a growth chamber (12 h light/12 h dark photoperiod, illumination 200 μmol m^−2^ s^−1^; 24 °C) for 13 days. Solutions were prepared in a phosphate buffer with the addition of 1 M of urea. [^3^H]hyaluronic acid of a final concentration of 30 g/L and specific radioactivity of 10 MBq/g was used. A part of around 1 cm of a leaf was partially immersed for one day in a 1 mL solution of tritium-labeled hyaluronic acid either with or without the addition of HA, as shown in Figure 6. One leaf of three plants was immersed in each solution. After 24 h, the immersed and non-immersed parts of the leaf were separately cut off from the plant, washed with pure water, dried with filter paper, and dried at 60 °C to a constant weight. Next, the leaf sections were dissolved in nitric acid, followed by radioactivity measurements.

### 3.3. Measuring Interfacial Tension and Contact Angle

Interfacial tension and contact angle were measured using the optical contact angle system OCA 15EC (DataPhysics Instruments GmbH, Filderstadt, Germany).

Interfacial tension at the aqueous–toluene interface was measured for all two-phase systems (Section 3.2) followed by radioactivity measurements. A 2 mL portion of the toluene phase was transferred to a quartz cuvette, and then a 5–6 μL drop of the aqueous phase was dispensed into the bulk toluene. The drop volume was chosen so that the drop would hang on the edge of the needle. The time-dependent interfacial tension was calculated from the shape of the drop using the DataPhysics software SCA 20 shape analysis software, 2017 (DataPhysics Instruments GmbH, Filderstadt, Germany) and the Young–Laplace equation as fast as possible regime during the 30 min.

Solutions of 30 mg/L of HA, 30 g/L of light hyaluronic acid, and an HA–hyaluronic acid mixture were prepared in a phosphate buffer containing 1 M of urea for contact angle of the leaf surface and solution measurements. A 4–5 μL drop was dispensed on the leaf surface and the wetting contact angle of the leaf surface and solution was measured using DataPhysics software in as fast a regime as possible during 15 min. The studies were performed at room temperature (20–25 °C) and normal humidity (45–55%). An independent experiment showed that under the experimental conditions, the evaporation of a 20 μL drop was close to 15 μL/h for. Therefore, during 15 min, the decrease in drop volume was controlled by both evaporation and adsorption. A wetting contact angle of 5–6 drops was measured for the first and second leaves of four plants.

### 3.4. Statistical Analysis

Mean values and standard deviations were calculated using Origin Pro 2016 software (OriginLab Corporation, Northampton, MA, USA). The average values in each repetition group were compared using a post hoc analysis of variance following a Kruskal–Wallis test. Datasets were compared by one-way analysis of variance (ANOVA) using the same software.

## 4. Conclusions

In conclusion, this study examines the effect of moisture-retaining compounds—hyaluronic acid and carboxymethyl cellulose—on the colloidal chemical properties of HA in an aqueous solution–organic liquid system. The findings demonstrate that HA exhibits optimal adsorption properties when the concentration of hyaluronic acid is 30 g/L, independent of its molecular weight. In contrast, the presence of CMC reduces the surface activity of HA.

The effectiveness of wheat leaf wetting and hyaluronic acid absorption has also been investigated. However, the presence of HA showed negligible effects on either hyaluronic acid absorption or its systemic distribution within the plant. The study demonstrated that a combined solution of HA and hyaluronic acids provides better wetting for both young and mature leaves compared to individual substance solutions. Such a synergistic effect should provide a more uniform coverage of leaves with fertilizers, which is especially important for hydrophobic crops. This will lead to reduced chemical consumption due to improved droplet spreading and retention. The purpose of future research will be to analyze the interaction between HA–carbohydrate composites and plants at the physiological level. We propose using natural polymers as adjuvants that could potentially replace synthetic surfactants in agriculture. From an environmental perspective, they are safer and multifunctional. However, while replacing synthetic surfactants with natural polymers is promising for sustainable agriculture, it requires optimizing formulations for specific crops and developing cost-effective production technologies.

## Figures and Tables

**Table 1:** The mass content of hyaluronic acid in the leaf after 24 h exposure in a solution of tritium-labeled hyaluronic acid either with or without the addition of humic acids. HA concentration is 30 mg/L, hyaluronic acid concentration is 30 g/L, pH is 6.8.

Solution Composition	Part of Leaf	Mass Content of Hyaluronic Acid, mg per 1 g of Leaf
[^3^H]Hyaluronic acid	immersed	82 ± 40
	non-immersed	2.3 ± 1.5
[^3^H]Hyaluronic acid + HA	immersed	112 ± 30
	non-immersed	1.8 ± 1.0

## Data Availability

Data are contained within the article.

## Abbreviations

The following abbreviations are used in this manuscript:

HA	Humic acids
CMC	Carboxymethyl cellulose
CA	Contact angle
